# A case for routine microbial diagnostics: Results from antimicrobial susceptibility testing in post-traumatic wound infections at a Ugandan tertiary care hospital

**DOI:** 10.1371/journal.pgph.0001880

**Published:** 2023-08-15

**Authors:** Laura Jung, James Kiwanuka, Leah Mbabazi, Vivian Nakate, Joseph Musaazi, Hawah Nabajja, Henry Kajumbula, Christoph Lübbert, Erisa Mwaka, Sara Nsibirwa, Amrei von Braun

**Affiliations:** 1 Department of Medicine I, Division of Infectious Diseases and Tropical Medicine, Leipzig University Medical Center, Leipzig, Germany; 2 Department of Orthopedics/Trauma, Mulago National Referral Hospital, Kampala, Uganda; 3 Infectious Diseases Institute, College of Health Sciences, Makerere University, Kampala, Uganda; 4 Department of Medical Microbiology, Makerere University, Kampala, Uganda; Malawi-Liverpool-Wellcome Trust Clinical Research Programme, MALAWI

## Abstract

The global spread of antimicrobial resistance (AMR) poses an increasing challenge for clinicians in Uganda, where microbiological diagnostics are not routinely available or accessible. The aim of this study was to determine pathogen prevalence and antibiotic resistance patterns in patients with wound infections following trauma at a national referral hospital in Kampala, Uganda. In addition, the suitability of currently used empirical treatment options in this setting was evaluated. This prospective, observational study analysed antimicrobial prescriptions, culture results and antimicrobial sensitivity testing (AST) of wound swabs and blood samples from patients with clinical signs of wound infections on the trauma ward. A total of 124 patients (n = 99, 79.8% male) with a median age of 30 years (IQR 23–39) were enrolled between October 2021 and January 2022. Wound infections were classified as nosocomial in 69% of the cases. Pathogens were isolated from 122 wound swabs, yielding 238 bacterial isolates. The most prevalent pathogens were gram-negative bacteria including *Escherichia coli* (n = 48, 20.2%) and *Acinetobacter* spp. (n = 43, 18.1%). Empiric treatment consisted of ceftriaxone and gentamicin which was administered to 67.2% (n = 78) and 62.1% (n = 72) of patients, respectively. High rates of antimicrobial resistance could be demonstrated across gram-negative and gram-positive species towards the most common empiric antibiotics. Following the AST results, over 95% (n = 111) of patients required a change of treatment. Our findings demonstrate that current empiric treatment for wound infections is missing its target in hospitalized patients in Kampala. To address the growing problem of AMR in Uganda, there is a pressing need to enhance diagnostic capacity and implement structured antimicrobial stewardship programs.

## 1. Introduction

Sometimes called a ‘silent pandemic,’ antimicrobial resistance (AMR) grows unattended in many regions of the world and increasingly threatens our ability to prevent and treat infections [1]. While Sub-Saharan Africa carries the highest mortality and disease burden [2], even basic microbiological diagnostics are still inaccessible in many health care facilities [3,4]. A review of AMR in the Eastern African Region showed elevated resistance rates especially towards the most commonly used antibiotics, ampicillin, cotrimoxazole and ceftriaxone [5]. Due to limited capacity for microbiologic diagnostics and thus a lack of local surveillance data in resource-limited settings, the choice of antibiotics is mostly empirical, and rarely based on bacterial susceptibility [5,6]. In the absence of diagnostic services a general use of non-targeted, broad-spectrum antimicrobials is encouraged [7], driving resistance in itself.

### 1.1 Antimicrobial resistance in Uganda

Despite being the first African country to implement a structured national AMR surveillance program in alignment with the WHO Global Antimicrobial Resistance Surveillance System (GLASS) in 2015 [8], the magnitude of AMR in Uganda remains largely unknown due to a lack of access to microbiological diagnostics. A National Action Plan (NAP) on AMR was published in 2018 [9] and set strategic objectives on surveillance, diagnostics and access to antimicrobials [10]. However, so far no national lab-based surveillance system for routine care has been established [3]. Data collection is incomplete, and comprehensive antimicrobial stewardship (AMS) is not routinely performed at Ugandan hospitals [11]. Expertise and capacity in the existing laboratories is often skewed towards HIV, malaria, tuberculosis, and most recently COVID-19, leaving other areas of microbiology less developed [12].

Even though Uganda offers free basic health care, patients are mostly required to pay out-of-pocket for microbiological diagnostics, which many cannot afford. The retrospective data available from sentinel surveillance sites show a high rate of resistance to commonly used antibiotics for gram-positive and gram-negative pathogens alike [8,13]. In a recent study focusing on surgical patients in Mulago National Referral Hospital (MNRH) an increasing trend in AMR was reported over a five year period [14].

One explanation for the increase of resistance could be the overuse of ceftriaxone, especially as peri-operative prophylaxis and through prolonged post-operative administration [15,16]. A recent study from Kampala also showed that up to one out of every two patients admitted to medical wards received a prescription of ceftriaxone [15]. Further gaps can be found in the lack of locally adapted empirical prescribing guidelines for antimicrobials and the low compliance of clinicians with the national clinical guidelines [16,17]. Documentation on drug administration is often not sufficient, leading to frequent missed doses of antimicrobial treatment even in hospital settings [18]. In addition, self-medication poses a problem, as many patients purchase antimicrobials directly over-the-counter (OTC) in community pharmacies or drug shops [19,20]. The lack of regulation on OTC antimicrobial purchases leads to a surveillance gap, increases the risk of mis- and overuse of antimicrobials, and subsequently the spread of AMR.

### 1.2 Wound infections after traumatic injuries

Road traffic accidents (RTA) are a major contributor to mortality and morbidity in Uganda accounting for over 12,000 deaths annually [21]. Motorcycle-users (so-called ‘boda-boda’ drivers) and pedestrians have the highest risk of severe or fatal injury [22]. High-energy trauma as experienced during RTAs, results in skin breaks, soft tissue damage and/or open fractures, which increases the risk of introduction of environmental contaminants and subsequent infections [23]. A recent study at a regional hospital in Uganda reported wound infections as the most common adverse event after motorcycle-related limb injuries [24]. Late presentation to the hospital after RTAs, scarcity of resources and delays of surgical treatment further contribute to high infection rates [25]. However, high quality microbial data on the issue is scarce and no data from Uganda is available. The pathogen prevalence data from open fractures in other tertiary hospitals in low resource settings suggest high rates of wound infection, with predominantly gram-negative bacteria (mainly *E*. *coli*, *P*. *aeruginosa*, *Klebsiella pneumoniae)* and a high potential for multi-drug resistance [26–28].

To address the growing concern of AMR in wound infections and the suitability of the current empiric treatment, this study aimed to determine pathogens and their resistance patterns among a population of hospitalized patients with wound infections following trauma. No previous assessment of pathogens and resistance profiles from trauma wards was available. We hypothesized that there is a high rate of resistance to first choice empirical treatment, and that a switch of antibiotic treatment would be necessary in these cases.

## 2. Materials and methods

### 2.1 Ethics statement

This study was reviewed and approved by the Mulago Hospital Research and Ethics Committee (MHREC 1835), and the Uganda National Council for Science and Technology (date of approval: June 18, 2020). Written informed consent was obtained from all participants prior to enrolment. For participants under 18 years of age, written informed consent was obtained from a parent or guardian. The funding agency had no role in study design, data collection, data analysis, data interpretation, or writing of this manuscript.Patients were compensated for their participation. Costs of diagnostics were covered by the study. Costs for reserve antibiotics were covered by the study in case patients were unable to afford them.

### 2.2 Study design & setting

This cross-sectional, observational study was performed in the Department of Orthopedics/Trauma of Mulago National Referral Hospital (MNRH) in Kampala, Uganda. The MNRH is the largest government hospital in Uganda and acts as a teaching hospital for Makerere University´s College of Health Sciences. The trauma ward consists of 75 in-patient beds. Up to three quarters of the patients are admitted with open musculoskeletal injuries due to road traffic accidents, or more rarely, open injuries due to falls and criminal violence. Due to high patient numbers and limited hospital resources microbiological identification of pathogens including susceptibility testing are not routinely available to patients on the ward.

### 2.3 Inclusion and exclusion criteria

Patients of all age groups on the MNRH orthopedic/trauma unit who developed clinical signs of wound infection (e.g. erythema /pus discharge) and/or fever were eligible for study participation. Potential participants were screened on the ward during daily ward rounds and/or dressing changes by a trained study nurse. Patients without clinical signs of wound infection but fever due to malaria or acute, untreated tuberculosis and patients participating in another study were excluded from the study.

### 2.4 Data and sample collection

Patients were screened by trained study staff (treating healthcare providers) when entering the trauma ward. Upon giving written informed consent eligible patients were consecutively enrolled on site. For the baseline assessment, information on demographics, past and present medical history including HIV status, details of reason for admission, as well as history of antibiotic treatment, was obtained. Urine pregnancy testing was done in female participants of child-bearing age (15–45 years). Blood samples were collected for general laboratory tests (complete blood count, creatinine, and aspartate aminotransferase) and two pairs of blood cultures. A malaria test was performed. Wound swabs were taken from all sites with clinical signs of infection by trained staff following the Levine method [29,30]. Empirical antimicrobial treatments, targeted treatments following microbiology testing and surgical treatments were documented. Wound infections were categorized as nosocomial according to WHO definitions: “A nosocomial infection is an infection occurring in a patient during the process of care in a hospital or other health-care facility which was not present or incubating at the time of admission” [31]. In-line with the cited WHO report, nosocomial infections included wound infections occurring > 48 hours after admission to the ward or at the site of surgery.

**Fig 1:**
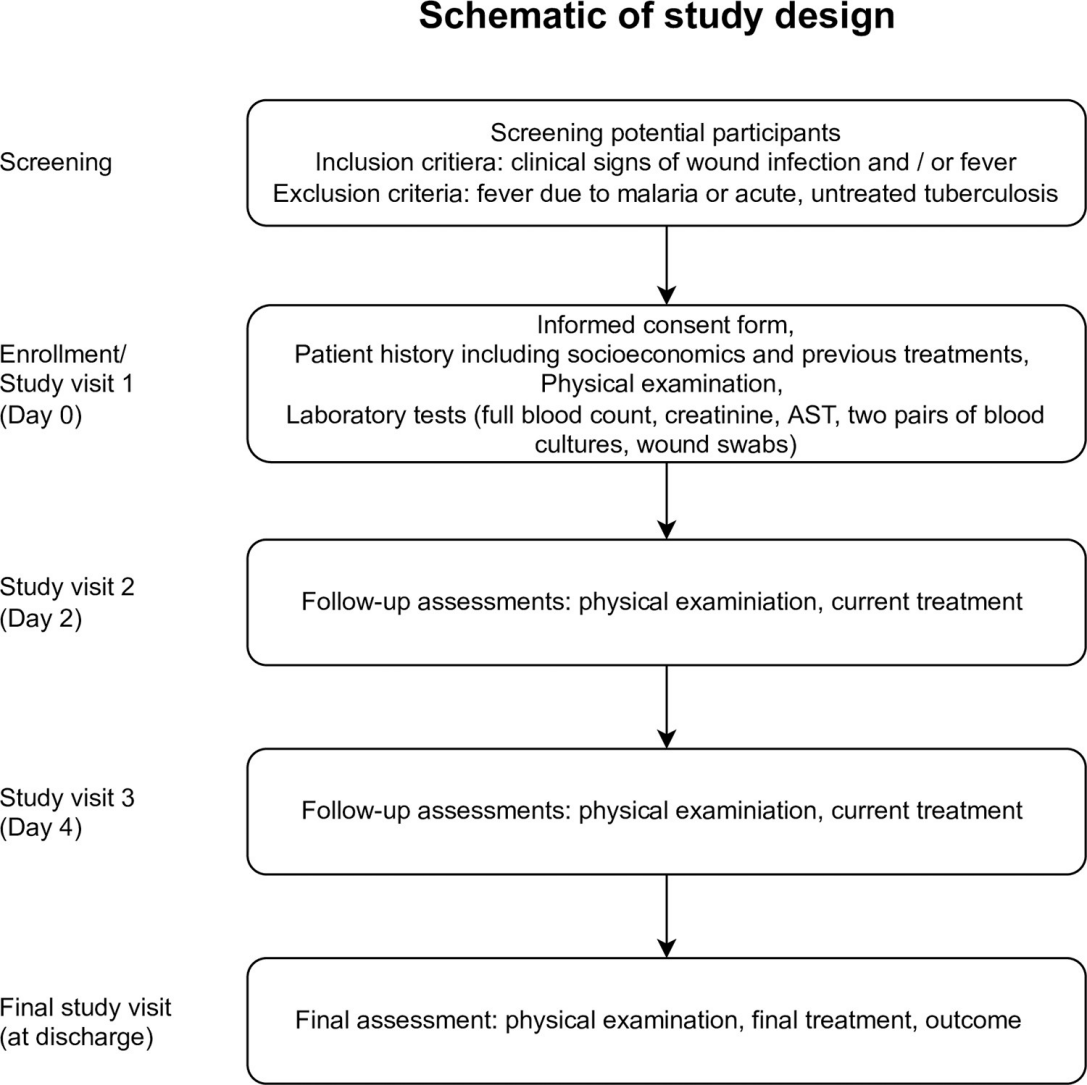
Schematic of study design.

Follow-up study visits were conducted two and four days after enrolment by the study staff on the ward. The final study visit was done upon discharge from the unit (full overview of study procedures in Fig 1). Clinical outcome was categorized as either complete recovery (limb function preserved), minor disability (joint stiffness requiring physiotherapy), moderate disability (soft tissue loss requiring cover, partial/complete digit amputations and minor compromise in function of the appendage), major disability (partial or complete loss of the affected limb with severe impairment of limb function requiring orthoses) or death.

### 2.5 Microbiological testing

Blood cultures were collected using BD Bactec Plus or in BD Bactec Peds aerobic bottles (Becton Dickinson, Franklin Lakes, NJ, USA). Paired wound swabs were collected on the ward using cotton tipped swabs with Amies transport media. Subsequently, specimens were sent to the Department of Microbiology at Makerere College of Health Sciences laboratories on the same campus for immediate analysis of bacterial growth and antimicrobial sensitivity. There, blood cultures were performed on a BACTEC9120 or FX40 system (Becton Dickinson, USA) and sub cultured onto Chocolate, Blood and MacConkey agar if positive. Wound swabs were inoculated onto Chocolate, Blood and MacConkey agar and incubated in a 5–7% Carbon dioxide incubator. Bacterial identification was done manually using colony morphology, gram stain characteristics and conventional biochemical tests according to the laboratory’s standard operating procedures and the automated BD Phoenix instrument (Becton Dickinson, USA), according to the manufacturer’s instructions. Antimicrobial susceptibility tests were performed using a Kirby-Bauer disc diffusion method based on the 2020 CLSI standards [32]. Extended-spectrum beta-lactamases (ESBLs) were detected by comparing zone sizes of two third generation cephalosporins alone versus two third generation cephalosporin/clavulanic acid combinations using disk diffusion [33].

### 2.6 Data management and statistical analysis

Data was collected and securely managed using data entry forms pre-programmed on the web-based software platform REDCap (Research Electronic Data Capture, https://projectredcap.org/) hosted at the Infectious Diseases Institute, College of Health Sciences, Makerere University. Data quality was ensured by adding validation checks in the REDCap database system. Data in the database was continuously checked and errors were corrected immediately. Data were exported into Microsoft Excel 2016 (Microsoft Corporation, Redmond, WA, USA) and analyzed in STATA version 6.1 (StataCorp, TX, USA). We described participants’ characteristics using frequencies and percentages for categorical variables. Prevalence of pathogens in our sample was estimated as a proportion of participants with positive cultures. Resistance profiles and distribution of pathogens by specific antibiotics were summarized using frequencies and proportions.

## 3. Results

### 3.1. Characteristics of study population

Out of 125 patients screened between October 2021 and January 2022, 124 patients (79.8% male) with a median age of 30 years (IQR: 23–40) consented to participation and were enrolled. The majority of patients was admitted with trauma due to road traffic incidents (n = 102, 82.3%). Population demographics and clinical characteristics are summarized in Table 1. The majority of participants had undergone surgery for their injuries (n = 112, 90.3%), which on average was performed 7 days (IQR 4–9) before enrolment. Similarly, most patients had been prescribed antimicrobial treatment before study inclusion (n = 116, 93.5%). A total of 69% (86/124) of the wound infections were classified as nosocomial.

**Table 1:** Demographic and clinical characteristics of study population (n = 124).

Characteristics		Number (%)
**Sex**	Male	99 (79.8)
	Female	25 (20.2)
**Age** **(in years)**	<15	7 (5.6)
	15–17	4 (3.2)
	18–24	28 (22.6)
	25–34	39 (31.5)
	35–49	31 (25.0)
	50+	15 (12.1)
**HIV status**	Negative	116 (93.5)
	Positive	7 (5.7)
	Not assessed	1 (0.8)
**Pre-existing condition** **(other than HIV and Diabetes mellitus)** ^**1**^	Yes	4 (3.2)
	No	120 (96.8)
**Diabetes mellitus**	Yes	4 (3.2)
	No	120 (96.8)
**Mechanism of injury**	Road traffic incident	102 (82.3)
	Violent assault incl. gunshot	9 (7.3)
	Other	13 (10.4)
	Fracture of lower limbs	100 (80.6)
	Fracture of upper limbs	15 (12.1)
**Type of trauma**	Other fracture	2 (1.6)
	Trauma without fractures	7 (5.7)
**Hospital stay before study enrolment (in days)**	Median	7
	IQR	5–11.25
**Surgical treatment** **before study inclusion**	Yes	112 (90.3)
	No	12 (9.7)
**Time since most recent surgical treatment (in days)** ^**2**^	Median	6
	IQR	4–9
**Type of surgical treatment** ^**2**^	Debridement	90 (80.4)
	External fixation	17 (15.2)
	Amputation	16 (14.3)
	Other^3^	14 (12.5)
**Antibiotic treatment** **before study inclusion**	Yes	116 (93.5)
	No	8 (6.5)
**Antibiotic treatment in the past year** ^**4**^	Yes	33 (26.6)
	No	91 (73.4)
**Source of antibiotics in the past year** ^**5**^	Drug shop/ pharmacy	27 (81.8)
	Hospital	4 (12.1)
	Health centre	1 (3.0)
	Unknown	1 (3.0)
**Antibiotic treatment in the past four weeks** ^**6**^	Yes	14 (11.3)
	No	110 (88.7)
**Source of antibiotics in the past four weeks** ^**7**^	Drug shop/ pharmacy	10 (71.4)
	Hospital	2 (14.3)
	Health centre	1 (7.1)
	Unknown	1 (7.1)

^1^ Including COPD/asthma, cerebrovascular disease and hypertension.

^2^ n = 112 (patients with surgical treatment), multiple surgical treatments possible.

^3^ Other surgical treatments: Fasciotomy (1), Wire osteosynthesis (2), Screw / plate osteosynthesis (4) Intramedually nailing (3), Achilles tendon repair (2), Incision and drainage (1), Plaster of Paris back slab (1), Skeletal traction (1).

^4^ Excluding the past four weeks.

^5^ n = 33.

^6^ Before hospital admission.

^7^ n = 14.

### 3.2 Type and prevalence of pathogens and resistance patterns

**Fig 2:**
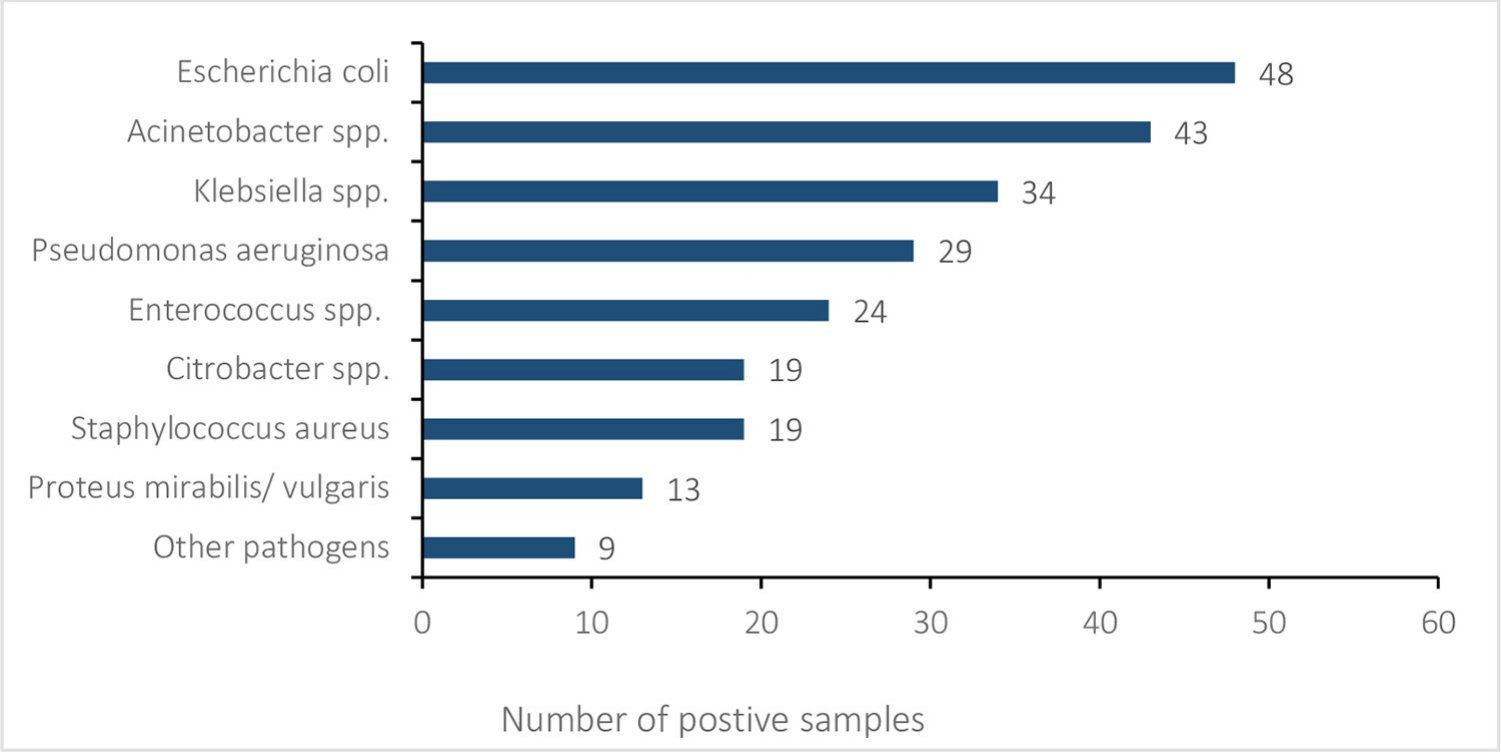
Type and frequencies of bacteria isolated from wound swabs. Other pathogens: *Enterobacter* spp., *Morganella morganii*, *Providencia* spp.

Pathogens were isolated from nearly all wound swabs (122/124). The majority (n = 83, 67.5%) showed mixed growth of two or more isolates, while 31.5% (n = 39) of swab cultures showed single-colony growth. In total, 238 pathogens were isolated from the 122 positive wound swabs. The most common pathogens were gram-negatives, mainly *Escherichia (E*.*) coli*, *Acinetobacter* spp., *Klebsiella* spp., *Pseudomonas (P*.*) aeruginosa*, followed by gram-positive *Enterococcus* spp. (Fig 2).

With 98% resistance towards ceftriaxone and 51% resistance towards gentamicin, a large proportion of all pathogens demonstrated resistance, or were intrinsically resistant, to the most commonly used empiric antibiotics (Table 2). Additionally, over 60% of *E*. *coli* isolates displayed resistance towards ciprofloxacin and over 40% towards piperacillin/tazobactam. Sensitivity was preserved for carbapenems (<5% resistance) and amikacin. However, *Acinetobacter* spp., the second-most common pathogen identified, displayed resistance towards carbapenems in 40% of isolates. In *P*. *aeruginosa* isolates intrinsically resistant to ceftriaxone, resistance towards ciprofloxacin was found in 15% and towards carbapenems in 25% of isolates. The majority (n = 17, 85%) of *Enterococcus* spp. isolates displayed vancomycin resistance. While *Staphylococcus aureus* was rarely isolated in our population (n = 19), four (21.1%) isolates were methicillin resistant. Overall, 45 isolates (18.9%) were ESBL producers. No relevant differences could be shown between nosocomial and community acquired infections with regard to most prevalent pathogens and resistance profiles (for details see S1 and S2 Tables).

**Table 2:** Most prevalent pathogens (n>20 isolates) and their resistance towards selected antimicrobials.

Isolates n/N (%)	*E*. *coli* N = 48	*Acinetobacter* spp. N = 43	*Klebsiella* spp. N = 34^1^	*P*. *aeruginosa* N = 28	*Enterococcus* spp. N = 24
**Ceftriaxone/Cefotaxime** ^**2**^	43/45 (95.7)	-^3^	30/32 (93.8)	-^3^	-^3^
**Gentamicin**	22/40 (55.0)	32/39 (82.1)	21/27 (77.8)	11/24 (45.8)	3/21 (14.3)^4^
**Ampicillin**	31/31 (100.0)	-^3^	-^6^	-^3^	3/11 (27.3)
**Amoxicillin+ clavulanic acid**	14/32 (43.7)	-^3^	5/18 (27.8)	-^3^	-^3^
**Cefepime**	8/8 (100.0)^5^	28/35 (80.0)	4/6 (66.7)^6^	5/21 (23.8)^6^	-
**Ciprofloxacin**	21/33 (63.6)	26/32 (81.3)	10/26 (38.5)	3/20 (15.0)	3/4 (75.0)
**Meropenem/Imipenem** ^**2**^	1/46 (2.2)	15/42 (35.7)	4/32 (12.5)	7/28 (25.0)	-^3^
**Amikacin**	2/21 (9.5)	3/33 (9.1)	2/12 (16.7)	7/17 (41.2)	-^3^
**Chloramphenicol**	11/41 (26.8)	-^3^	12/30 (40.0)	-^3^	4/18 (22.2)
**Piperacillin/** **Tazobactam**	10/23 (43.5)	29/36 (74.42)	9/18 (50.0)	4/23 (17.4)	-

^1^ K*lebsiella oxytoca* (n = 4) and *Klebsiella pneumonia* (n = 30).

^2^ One out of two was set.

^3^ Intrinsic resistance, not tested.

^4^High-level gentamicin resistance.

^5^ Set in case of resistance to 3rd generation cephalosporins.

^6^ Intrinsic resistance.

Of the blood cultures obtained, only 6 out of 124 pairs flagged positive, containing seven different pathogens (*E*. *coli* 3/6, *Enterococcus* spp. 3/6, *Morganella morganii* 1/6). Fungal infections were not detected in wound swabs or blood cultures.

### 3.3. Antibiotic prescriptions

At the time of study inclusion, the majority of patients (n = 116, 93.6%) were already receiving an antimicrobial treatment–either from MNRHs Casualties’ Department or other health care providers / pharmacies outside of MNRH. The most common empiric treatments were ceftriaxone (n = 78/116, 67.2%) and gentamicin (n = 72/116, 62.1%), followed by levofloxacin (n = 25/116, 21.6%) and metronidazole (n = 11/116, 9.5%). Twenty-six participants received monotherapy, while 90 (78%) received a combination of at least two antimicrobials, resulting in a total of 211 antimicrobial administrations in 116 patients.

**Fig 3:**
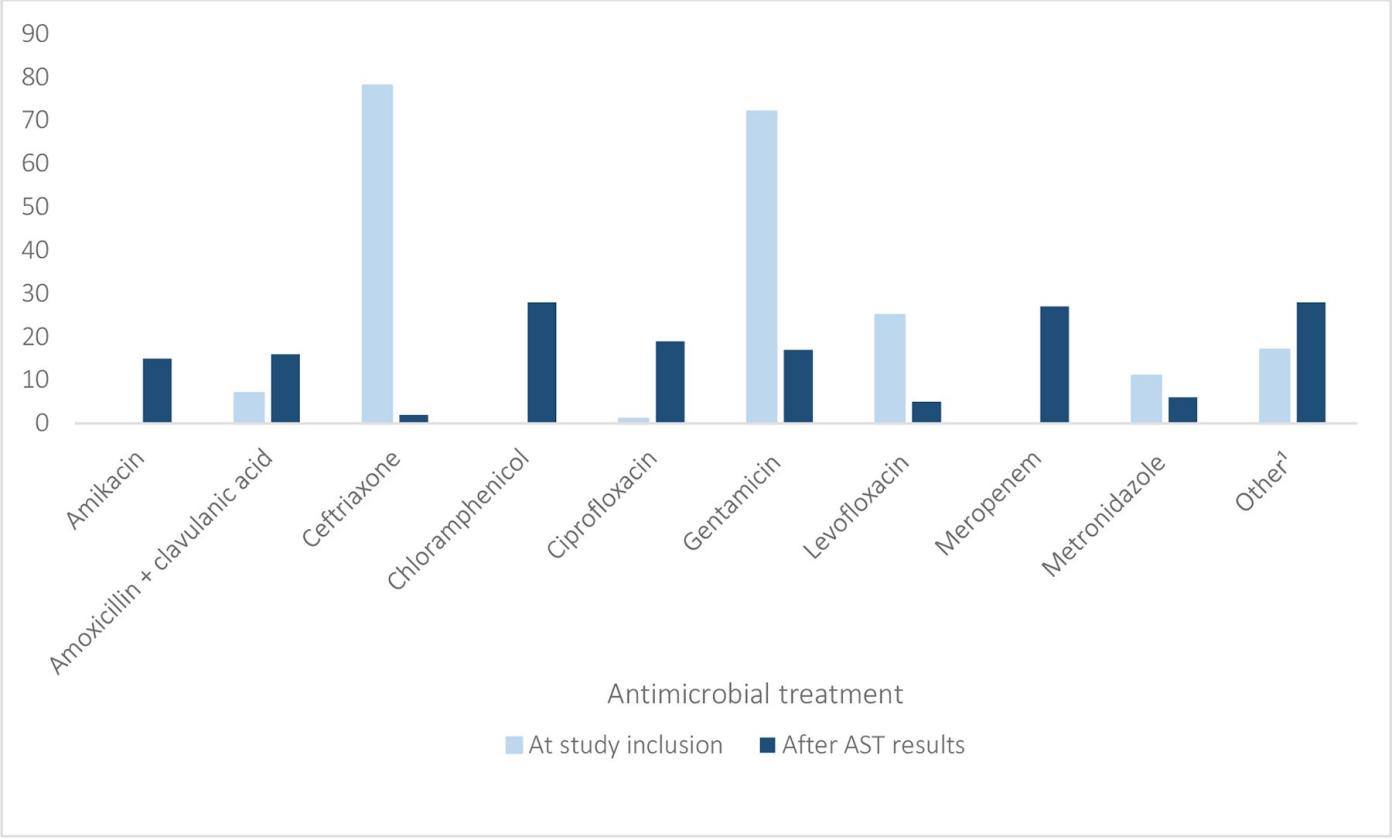
Antimicrobial agents prescribed to patients at time of study inclusion (n = 211 antimicrobial administrations in 116 patients) and after AST results were received (n = 163 antimicrobial administrations in 124 patients). ^1^ Other locally available antimicrobials prescribed: cefixime, *Flucamox* (amoxicillin + flucloxacillin), linezolid, ornidazole, *Sulbactox* (ceftriaxone + sulbactam).

Upon review of AST results, the majority of patients (n = 111, 95.7%) required a change of antimicrobial treatment (Fig 3). In our sample, microbiological diagnostics led to a diversification of treatments, with chloramphenicol (n = 28/124, 22.6%), meropenem (n = 27/124, 21.8%) and ciprofloxacin (n = 19/124, 15.3%) being the most commonly used agents after receiving AST results.

### 3.4 Clinical outcomes

The median length of stay on the ward was 22 days (IQR 15–41 days). Twelve patients (9.7%) recovered completely by the time of discharge (Table 3). The majority (89.5%) was left with a disability, and a 23-year-old male patient died intra-operatively due to cardiac arrest. None of the participants were lost to follow-up.

**Table 3:** Clinical outcomes of study participants (n = 124).

Outcome		Number (%)
**Clinical outcome at** **discharge**	Complete recovery	12 (9.7%)
	Minor disability	62 (50.0%)
	Moderate disability	28 (22.6%)
	Major disability	21 (16.9%)
	Death	1 (0.8%)

## 4. Discussion

We report here the clinical and microbiological findings of 124 patients admitted to the largest orthopedic/trauma unit in Uganda with wound infections following trauma. To the best of our knowledge, this is the first prospective clinical study on the type, prevalence and resistance patterns of pathogens from wound infections in a Ugandan national referral hospital. The data presented highlights that pathogens resistant to first-choice empiric treatments are highly prevalent in the study population, and is therefore of high clinical significance for trauma care in Uganda.

RTAs are one of the most common cause for severe trauma in Uganda, with men being three times more likely than women to suffer from RTA injuries and being hospitalised. Sex and age composition in our sample, does not only correspond to RTA victim statistics [22,34,35], but is also comparable to other studies from surgical wards in Uganda [35,36]. The HIV seroprevalence of 5.7% in our sample is in accordance with the Ugandan average (2020 prevalence estimates: 5.4% among adults aged 15–49 years [37]).

Healthcare-associated infections, especially surgical-site infections are more common in low resource settings [38]. In our study, more than two-thirds of wound infections were classified as nosocomial, which is also reflected by the pathogens identified. Most swabs yielded gram-negative pathogens, predominantly *E*. *coli* and *Acinetobacter* spp.. These results corresponds to data of trauma wards in other low resource settings, which show similar pathogen distributions in wound infections [26–28,39]. Of the five most common pathogens in our sample, three were intrinsically resistant to the most commonly used empiric treatment ceftriaxone (*Acinetobacter* spp., *P*. *aeruginosa* and *Enterococcus* spp.), and the remaining species displayed resistance to ceftriaxone in over 90% (*E*. *coli* 95.7%, *Klebsiella* spp. 93.8%).

These high rates of resistances against commonly used empiric antibiotics are consistent with results of the few available studies from Uganda that focused on surgical patients, including patients with chronic wounds [40], patients with surgical site infections (SSI) [41], and a retrospective analysis of various diagnostic materials from MNRH over a period of five years [14]. A study on patients with SSI across several surgical wards of MNRH in 2011 showed lower rates of resistance, e.g. 78% resistance for *E*. *coli* towards ceftriaxone Carbapenem resistance of *Acinetobacter spp* in Uganda tertiary hospitals was previously estimated to lie between 4–24% depending on the study [42,43]. However, our analysis showed a resistance rate of 38%, which is a relevant increase. While the available data may be insufficient to determine a trend, it does support the need for accurate annual surveillance data from routine diagnostics to monitor the spread of AMR in the country and identify risk factors. Nosocomial infections might be more common in Uganda as hospital stays tend to be long due to treatment delays and IPC is often not optimal [44]. Despite the young age and low rate of comorbidities, a quarter of our study participants had used antimicrobials in the past year. Over 80% of them bought antimicrobials directly in community drug shops or pharmacies without a medical prescription, which underlines the high prevalence of self-prescription in our sample, similar to the available literature [19,20].

In our study, AST results led to a treatment switch for nearly all patients. Prior to our research intervention, empirical antibiotic treatment on the ward was commonly based on clinical judgement without knowledge of pathogen and resistance patterns. The ability to perform microbial testing was limited, since it is an unaffordable out-of-pocket expense for most patients in this setting. However, the data presented here suggests that empiric treatment with ceftriaxone and gentamicin misses the target when it comes to wound infections following trauma and can therefore not be recommend. The use of AST in our study enabled the diversification of treatment options and guided clinical decision-making. While some patients were in need of expensive, often hardly accessible carbapenems, in many cases cheap alternatives existed. In general, our results highlight that a setting with a high rate of nosocomial infections in trauma patients calls for antibiotics which have a high efficacy in the gram-negative spectrum, secure a high degree of tissue penetration and are easily accessible. However, our results also show that there was no “one-fits-all” empiric therapy for in-patients with wound infections. Instead, our results underline the need for targeted therapy based on AST. To address the insufficient treatment of patients and the spread of AMR on Uganda trauma wards, routine diagnostic as well as clinical treatment guidelines, which consider local resistance patterns, need to be established.

Commonly under-recognized, accessibility of diagnostics is fundamental to quality health care and should be incorporated in universal health coverage [7,45]. Both, microbial cultures and AST are part of the WHO list of essential in vitro diagnostics [46]. Timely results from AST need to be available to health care workers, and should form the foundation for AMS-related educational as well as audit-and-feedback interventions. The implementation of diagnostic infrastructure and antimicrobial stewardship (AMS) is not cheap [47] and involves a range of different building stones [48]. However, it might be cost-effective, considering the high price associated with rising AMR rates and the subsequent morbidity and mortality [7,49]. Diagnostic services therefore need to be included into publically funded health care and not be dependent on patients’ ability to pay. To enable low-resource settings like Uganda to provide more diagnostic services, reasonably priced consumables, laboratory management systems and reliable supply chains are required.

## 5. Strength and limitations

Compared to previous studies using routine or retrospective data from surgical wards, this study’s prospective design serves as an important advantage [14]. It included all patients with clinical signs of wound infections, whereas the routine data is skewed towards patients who are able to pay for diagnostics. Due to the set-up of study procedures embedded the ward, there was no loss to follow-up. Thus, this study delivers an accurate picture of the distribution of pathogens and their resistance profiles in wound infections on the trauma ward. As described above, the study populations composition regarding sex, age and HIV-prevalence, suggests a sufficient representability for trauma patients in Ugandan tertiary care centres. The study was set in the clinical care setting and used the same pathways as routine care, the results therefore mirror the real-life situation on the ward.

However, our study also has some limitations, which are partly due to the monocentric design with a limited number of participants (n = 124). Baseline data, including prior treatments and in-hospital stays, was collected through patient interviews. This potentially led to recall bias, especially concerning events in the past. However, there are no alternative data collection systems for this information. Because of incomplete documentation of referrals and self-treatment with over-the-counter antibiotics, the timing and dose of previous treatments and the duration of previous hospitalizations were not available for assessment. In this respect, a mixed picture of predominantly nosocomial, possibly (still ambulatory-acquired) and fuzzy "in-between" situations must be accepted. Since this was an observational study, it is not possible to compare clinical outcomes of our study participants for instance to a matched population. Furthermore, the majority of patients received antimicrobial treatment before the study samples could be collected, which may have contributed to the low number of positive blood cultures and a pre-selected bacterial spectrum of the wound swabs.

As wounds following trauma are a non-sterile environment, and contaminants or skin-colonizing isolates might be sampled, results from wound swabs should be interpreted with caution [50,51]. Due to the study setting on the ward, it was not possible to obtain deeper cultures (e.g. intraoperative sampling) to guide therapy in this context. Instead, superficial cultures were obtained which may not represent the infectious pathogens. In order to optimize sample collection, the study teams were trained in appropriate sampling according to the Levine method [29,30]. These swabs still may identify bacteria superficially colonizing the wounds. As no swabs identified Group A *Streptococcus* and few identified *Staphylococcus aureus*, we would not assume that these bacteria represent the infectious causes. As most samples were taken after antibiotics, we would expect to see higher rates of resistance among colonizing bacteria as well. However, this does give a snapshot which bacteria are identified in and around patients, pointing to increasing resistance. In particular, *Acinetobacter* spp. infections show high levels of resistance. To add more information on pathogens in wound infections, intraoperative samples and samples taken before the start of antibiotic therapy should be analysed in future studies.

Due to laboratory constraints some pathogens were not identified to genus level, including *Enterococcus* spp. and *Acinetobacter* spp., and ESBL production could unfortunately not be confirmed by a reference method. It should be considered that due to the urban setting, RTA was the main reason for trauma in our sample, and the results might not be representative for health care settings where other sources of trauma overweigh.

## 6. Conclusions

While no “one-fits all” solution for antimicrobial treatment of wound infections among in-patients following high-energy trauma can be recommended based on our results, our findings highlight the need to rethink current empiric treatment and expand the use of microbiological diagnostics in this setting. Furthermore, our results add further data to the existing body of research documenting the concerning trend of increasing AMR in the country. The substantial consumption of antimicrobials without prior diagnostics, as highlighted in this study, exacerbates the risk of rising AMR. In order to improve patient care directly and halt the further spread of AMR in the region, access to routine pathogen identification and antimicrobial susceptibility testing is urgently needed.

## Supporting information

S1 Table Most prevalent pathogens and their resistance towards selected antimicrobials for nosocomial infection subgroup. (DOCX)

S2 Table Most prevalent pathogens and their resistance towards selected antimicrobials for community acquired infection subgroup. (DOCX)

## References

[1] World Health Organization. Antimicrobial resistance Factsheet. 2021 [updated 11 Jul 2022; cited 21 Jul 2022]. Available from: https://www.who.int/news-room/fact-sheets/detail/antimicrobial-resistance.

[2] Murray CJL, Ikuta KS, Sharara F, Swetschinski L, Robles Aguilar G, Gray A, et al. Global burden of bacterial antimicrobial resistance in 2019: a systematic analysis. The Lancet. 2022; 399:629–55. doi: 10.1016/S0140-6736(21)02724-0 35065702 PMC8841637

[3] Chaplain D, Asutaku BB, Mona M, Bulafu D, Aruhomukama D. The need to improve antimicrobial susceptibility testing capacity in Ugandan health facilities: insights from a surveillance primer. Antimicrob Resist Infect Control. 2022; 11:23. Epub 2022/02/03. doi: 10.1186/s13756-022-01072-4 . 35115045 PMC8812180

[4] World Health Organization. Regional Office for Africa. Antimicrobial resistance in the WHO African Region: a systematic literature review.; 2021.

[5] Ampaire L, Muhindo A, Orikiriza P, Mwanga-Amumpaire J, Bebell L, Boum Y. A review of antimicrobial resistance in East Africa. Afr J Lab Med. 2016; 5:432. Epub 2016/09/15. doi: 10.4102/ajlm.v5i1.432 . 28879114 PMC5436405

[6] Ronat J-B, Natale A, Kesteman T, Andremont A, Elamin W, Hardy L, et al. AMR in low-resource settings: Médecins Sans Frontières bridges surveillance gaps by developing a turnkey solution, the Mini-Lab. Clin Microbiol Infect. 2021; 27:1414–21. doi: 10.1016/j.cmi.2021.04.015 . 33932617

[7] Fleming KA, Horton S, Wilson ML, Atun R, DeStigter K, Flanigan J, et al. The Lancet Commission on diagnostics: transforming access to diagnostics. The Lancet. 2021; 398:1997–2050. doi: 10.1016/S0140-6736(21)00673-5 . 34626542 PMC8494468

[8] Nabadda S, Kakooza F, Kiggundu R, Walwema R, Bazira J, Mayito J, et al. Implementation of the World Health Organization Global Antimicrobial Resistance Surveillance System in Uganda, 2015–2020: Mixed-Methods Study Using National Surveillance Data. JMIR Public Health Surveill. 2021; 7:e29954. Epub 2021/10/21. doi: 10.2196/29954 . 34673531 PMC8569544

[9] Government of Uganda. Antimicrobial Resistance National Action Plan. 2018–2023.

[10] Mugerwa I, Nabadda SN, Midega J, Guma C, Kalyesubula S, Muwonge A. Antimicrobial Resistance Situational Analysis 2019–2020: Design and Performance for Human Health Surveillance in Uganda. Trop Med Infect Dis. 2021; 6. doi: 10.3390/tropicalmed6040178 . 34698282 PMC8544686

[11] Kimbowa IM, Ocan M, Eriksen J, Nakafeero M, Obua C, Stålsby Lundborg C, et al. Characteristics of antimicrobial stewardship programmes in hospitals of Uganda. PLoS One. 2022; 17:e0268032. Epub 2022/05/10. doi: 10.1371/journal.pone.0268032 . 35536856 PMC9089898

[12] Kiwanuka SN, Namuhani N, Akulume M, Kalyesubula S, Bazeyo W, Kisakye AN. Uganda’s laboratory human resource in the era of global health initiatives: experiences, constraints and opportunities-an assessment of 100 facilities. Hum Resour Health. 2020; 18:13. Epub 2020/02/18. doi: 10.1186/s12960-020-0454-5 . 32070361 PMC7029471

[13] Obakiro SB, Kiyimba K, Paasi G, Napyo A, Anthierens S, Waako P, et al. Prevalence of antibiotic-resistant bacteria among patients in two tertiary hospitals in Eastern Uganda. Journal of Global Antimicrobial Resistance. 2021; 25:82–6. doi: 10.1016/j.jgar.2021.02.021 33662642

[14] Mboowa G, Aruhomukama D, Sserwadda I, Kitutu FE, Davtyan H, Owiti P, et al. Increasing Antimicrobial Resistance in Surgical Wards at Mulago National Referral Hospital, Uganda, from 2014 to 2018-Cause for Concern. Trop Med Infect Dis. 2021; 6. Epub 2021/05/19. doi: 10.3390/tropicalmed6020082 . 34069345 PMC8163195

[15] Kizito M, Lalitha R, Kajumbula H, Ssenyonga R, Muyanja D, Byakika-Kibwika P. Antibiotic Prevalence Study and Factors Influencing Prescription of WHO Watch Category Antibiotic Ceftriaxone in a Tertiary Care Private Not for Profit Hospital in Uganda. Antibiotics (Basel). 2021; 10. doi: 10.3390/antibiotics10101167 . 34680748 PMC8532977

[16] Kiggundu R, Wittenauer R, Waswa JP, Nakambale HN, Kitutu FE, Murungi M, et al. Point Prevalence Survey of Antibiotic Use across 13 Hospitals in Uganda. Antibiotics (Basel). 2022; 11. Epub 2022/02/04. doi: 10.3390/antibiotics11020199 . 35203802 PMC8868487

[17] Kagoya EK, van Royen K, Waako P, van Royen P, Iramiot JS, Obakiro SB, et al. Experiences and views of healthcare professionals on the prescription of antibiotics in Eastern Uganda: A qualitative study. Journal of Global Antimicrobial Resistance. 2021; 25:66–71. doi: 10.1016/j.jgar.2021.02.019 33667701

[18] Kiguba R, Karamagi C, Bird SM. Extensive antibiotic prescription rate among hospitalized patients in Uganda: but with frequent missed-dose days. J Antimicrob Chemother. 2016; 71:1697–706. doi: 10.1093/jac/dkw025 . 26945712 PMC4867101

[19] Mukonzo JK, Namuwenge PM, Okure G, Mwesige B, Namusisi OK, Mukanga D. Over-the-counter suboptimal dispensing of antibiotics in Uganda. J Multidiscip Healthc. 2013; 6:303–10. doi: 10.2147/JMDH.S49075 . 23990728 PMC3753154

[20] Mbonye AK, Buregyeya E, Rutebemberwa E, Clarke SE, Lal S, Hansen KS, et al. Prescription for antibiotics at drug shops and strategies to improve quality of care and patient safety: a cross-sectional survey in the private sector in Uganda. BMJ Open. 2016; 6:e010632. doi: 10.1136/bmjopen-2015-010632 . 26980439 PMC4800164

[21] World Health Organization. Global status report on road safety 2018. Geneva; 2018.

[22] Muni KM, Ningwa A, Osuret J, Zziwa EB, Namatovu S, Biribawa C, et al. Estimating the burden of road traffic crashes in Uganda using police and health sector data sources. Inj Prev. 2020. doi: 10.1136/injuryprev-2020-043654 . 32229535

[23] Garner MR, Sethuraman SA, Schade MA, Boateng H. Antibiotic Prophylaxis in Open Fractures: Evidence, Evolving Issues, and Recommendations. J Am Acad Orthop Surg. 2020; 28:309–15. doi: 10.5435/JAAOS-D-18-00193 . 31851021

[24] Okullo GO, Flores MJ, Peck CJ, Socci AR, Kisitu DK. Adverse events in the treatment of motorcycle-related isolated limb injuries at a regional hospital in Uganda: a prospective clinical analysis. Int Orthop. 2022; 46:71–7. doi: 10.1007/s00264-021-05060-y . 34296324

[25] Odatuwa-Omagbemi DO. Open fractures: epidemiological pattern, initial management and challenges in a sub-urban teaching hospital in Nigeria. Pan Afr Med J. 2019; 33:234. doi: 10.11604/pamj.2019.33.234.18141 . 31692766 PMC6814931

[26] Islam MS, Islam SS, Parvin S, Manjur M, Islam MR, Halder RC, et al. Current pathogens infecting open fracture tibia and their antibiotic susceptibility at a tertiary care teaching hospital in South East Asia. Infect Prev Pract. 2022; 4:100205. doi: 10.1016/j.infpip.2022.100205 . 35243317 PMC8857645

[27] Bediako-Bowan AAA, Kurtzhals JAL, Mølbak K, Labi A-K, Owusu E, Newman MJ. High rates of multi-drug resistant gram-negative organisms associated with surgical site infections in a teaching hospital in Ghana. BMC Infect Dis. 2020; 20:890. doi: 10.1186/s12879-020-05631-1 . 33238903 PMC7689982

[28] Raouf M, Ghazal T, Kassem M, Agamya A, Amer A. Surveillance of surgical-site infections and antimicrobial resistance patterns in a tertiary hospital in Alexandria, Egypt. J Infect Dev Ctries. 2020; 14:277–83. doi: 10.3855/jidc.12124 . 32235088

[29] Angel DE, Lloyd P, Carville K, Santamaria N. The clinical efficacy of two semi-quantitative wound-swabbing techniques in identifying the causative organism(s) in infected cutaneous wounds. Int Wound J. 2011; 8:176–85. doi: 10.1111/j.1742-481X.2010.00765.x . 21303456 PMC7950681

[30] Levine NS, Lindberg RB, Mason AD, JR, Pruitt BA, JR. The quantitative swab culture and smear: A quick, simple method for determining the number of viable aerobic bacteria on open wounds. J Trauma. 1976; 16:89–94. 1255833

[31] World Health Organization. Report on the Burden of Endemic Health Care-Associated Infection Worldwide. Geneva; 2011.

[32] CLSI. Performance Standards for Antimicrobial Susceptibility Testing. 30th ed.; 2020.

[33] Giriyapur RS, Nandihal NW, Krishna BVS, Patil AB, Chandrasekhar MR. Comparison of disc diffusion methods for the detection of extended-spectrum Beta lactamase-producing enterobacteriaceae. J Lab Physicians. 2011; 3:33–6. doi: 10.4103/0974-2727.78561 . 21701661 PMC3118054

[34] Temizel S, Wunderlich R, Leifels M. Characteristics and Injury Patterns of Road Traffic Injuries in Urban and Rural Uganda-A Retrospective Medical Record Review Study in Two Hospitals. Int J Environ Res Public Health. 2021; 18. doi: 10.3390/ijerph18147663 . 34300111 PMC8304504

[35] Siya A, Ssentongo B, Abila DB, Kato AM, Onyuth H, Mutekanga D, et al. Perceived factors associated with boda-boda (motorcycle) accidents in Kampala, Uganda. Traffic Inj Prev. 2019; 20:S133–S136. Epub 2019/10/02. doi: 10.1080/15389588.2019.1658084 . 31577452

[36] Lubega A, Joel B, Justina Lucy N. Incidence and Etiology of Surgical Site Infections among Emergency Postoperative Patients in Mbarara Regional Referral Hospital, South Western Uganda. Surg Res Pract. 2017; 2017:6365172. Epub 2017/01/12. doi: 10.1155/2017/6365172 . 28168215 PMC5266862

[37] Uganda AIDS Commission Secreteriat. 2021 Factsheet -Facts on HIV and AIDS in Uganda 2021.; 2022.

[38] Abubakar U, Amir O, Rodríguez-Baño J. Healthcare-associated infections in Africa: a systematic review and meta-analysis of point prevalence studies. J Pharm Policy Pract. 2022; 15:99. doi: 10.1186/s40545-022-00500-5 . 36494700 PMC9733066

[39] Lakoh S, Le Yi, Russell JBW, Zhang J, Sevalie S, Zhao Y, et al. The burden of surgical site infections and related antibiotic resistance in two geographic regions of Sierra Leone: a prospective study. Ther Adv Infect Dis. 2022; 9:20499361221135128. doi: 10.1177/20499361221135128 . 36518726 PMC9742716

[40] Khalim W, Mwesigye J, Tungotyo M, Twinomujuni SS. Resistance pattern of infected chronic wound isolates and factors associated with bacterial resistance to third generation cephalosporins at Mbarara Regional Referral Hospital, Uganda. PLoS One. 2021; 16:e0261264. Epub 2021/12/16. doi: 10.1371/journal.pone.0261264 . 34914757 PMC8675733

[41] Hope D, Ampaire L, Oyet C, Muwanguzi E, Twizerimana H, Apecu RO. Antimicrobial resistance in pathogenic aerobic bacteria causing surgical site infections in Mbarara regional referral hospital, Southwestern Uganda. Sci Rep. 2019; 9:17299. Epub 2019/11/21. doi: 10.1038/s41598-019-53712-2 . 31754237 PMC6872727

[42] Seni J, Najjuka CF, Kateete DP, Makobore P, Joloba ML, Kajumbula H, et al. Antimicrobial resistance in hospitalized surgical patients: a silently emerging public health concern in Uganda. BMC Res Notes. 2013; 6:298. Epub 2013/07/27. doi: 10.1186/1756-0500-6-298 . 23890206 PMC3729663

[43] Kateete DP, Nakanjako R, Namugenyi J, Erume J, Joloba ML, Najjuka CF. Carbapenem resistant Pseudomonas aeruginosa and Acinetobacter baumannii at Mulago Hospital in Kampala, Uganda (2007–2009). Springerplus. 2016; 5:1308. doi: 10.1186/s40064-016-2986-7 . 27547682 PMC4978656

[44] Opollo MS, Otim TC, Kizito W, Thekkur P, Kumar AMV, Kitutu FE, et al. Infection Prevention and Control at Lira University Hospital, Uganda: More Needs to Be Done. Trop Med Infect Dis. 2021; 6. doi: 10.3390/tropicalmed6020069 34062871 PMC8167580

[45] Pai M, Walia K, Boehme CC. Essential medicines and essential diagnostics: a package deal. Lancet Public Health. 2019; 4:e492. doi: 10.1016/S2468-2667(19)30165-3 . 31451443

[46] World Health Organization. First WHO model list of essential in vitro diagnostics.; 2019.

[47] Roberts T, Luangasanatip N, Ling CL, Hopkins J, Jaksuwan R, Lubell Y, et al. Antimicrobial resistance detection in Southeast Asian hospitals is critically important from both patient and societal perspectives, but what is its cost. PLOS Global Public Health. 2021; 1:e0000018. doi: 10.1371/journal.pgph.0000018 34746931 PMC7611947

[48] Jacobs J, Hardy L, Semret M, Lunguya O, Phe T, Affolabi D, et al. Diagnostic Bacteriology in District Hospitals in Sub-Saharan Africa: At the Forefront of the Containment of Antimicrobial Resistance. Front Med (Lausanne). 2019; 6:205. doi: 10.3389/fmed.2019.00205 . 31608280 PMC6771306

[49] Gebretekle GB, Mariam DH, Mac S, Abebe W, Alemayehu T, Degu WA, et al. Cost-utility analysis of antimicrobial stewardship programme at a tertiary teaching hospital in Ethiopia. BMJ Open. 2021; 11:e047515. Epub 2021/12/17. doi: 10.1136/bmjopen-2020-047515 . 34921071 PMC8685939

[50] Chakraborti C, Le C, Yanofsky A. Sensitivity of superficial cultures in lower extremity wounds. J Hosp Med. 2010; 5:415–20. doi: 10.1002/jhm.688 . 20845440

[51] Spear M. Best technique for obtaining wound cultures. Plast Surg Nurs. 2012; 32:34–6. doi: 10.1097/PSN.0b013e31824a7e53 . 22395174

