# Correction: A case for routine microbial diagnostics: Results from antimicrobial susceptibility testing in post-traumatic wound infections at a Ugandan tertiary care hospital

**DOI:** 10.1371/journal.pgph.0004082

**Published:** 2024-12-09

**Authors:** The PLOS Global Public Health Staff

## Notice of Republication

This article was republished on October 30, 2023, to correct the name of the Editor. The publisher apologizes for the errors. Please download this article again to view the correct version. The originally published, uncorrected article and the republished, corrected articles are provided here for reference.

## Supporting information

S1 File Originally published, uncorrected article. (PDF)

S2 File Republished, corrected article. (PDF)
