# Supplementary material for: A case for routine microbial diagnostics: Results from antimicrobial susceptibility testing in post-traumatic wound infections at a Ugandan tertiary care hospital
Source: PLOS Glob Public Health. 2023 Aug 15;3(8):e0001880. doi: 10.1371/journal.pgph.0001880 (PMC10427013; doi:10.1371/journal.pgph.0001880)
Supplement: S1 Table — (DOCX) [file pgph.0001880.s001.docx]

S1 Table: Most prevalent pathogens and their resistance towards selected antimicrobials for nosocomial infection subgroup

| **Isolates**  **n/N (%)** | ***E. coli***  **N= 36** | ***Acinetobacter* spp.**  **N=29** | ***Klebsiella* spp.**  **N =23** | ***P. aeruginosa***  **N= 19** | ***Enterococcus* spp.**  **N= 12** |
| --- | --- | --- | --- | --- | --- |
| **Ceftriaxone/Cefotaxime^1^** | 33/34 (97.1) | 6/6 (100.0) | 20/22 (90.9) | -^2^ | -^2^ |
| **Gentamicin** | 18/31 (58.1) | 21/27 (77.8) | 12/17 (70.6) | 7/18 (38.9) | 2/11 (18.2)^3^ |
| **Ampicillin** | 22/22 (100.0) | -^2^ | -^5^ | -^2^ | 2/6 (33.3) |
| **Amoxicillin+ clavulanic acid** | 10/23 (43.5) | -^2^ | 2/10 (20.0) | -^2^ | -^2^ |
| **Cefepime** | 6/6 (100.0)^4^ | 17/24 (70.8) | 2/4 (50.0)^6^ | 5/16 (31.3)^4^ | -^2^ |
| **Ciprofloxacin** | 18/27 (66.7) | 18/22 (81.8) | 8/16 (50.0) | 2/14 (14.3) | 1/1 (100.0) |
| **Meropenem/Imipenem^1^** | -^2^ | 9/28 (32.1) | 2/22 (9.1) | 4/18 (22.2) | -^2^ |
| **Amikacin** | 2/16 (12.5) | 1/21 (4.8) | 1/10 (10.0) | 4/12 (33.3) | -^2^ |
| **Chloramphenicol** | 9/32 (28.1) | -^2^ | 8/20 (40.0) | -^2^ | 2/10 (20.0) |
| **Piperacillin/**  **Tazobactam** | 7/17 (41.2) | 20/25 (80.0) | 6/12 (50.0) | 2/17 (11.8) | -^2^ |

^1^ One out of two was set

^2^ Intrinsic resistance, not tested

^3^ High-level gentamicin resistance
^4^ Set in case of resistance to 3rd generation cephalosporins

^5^ Intrinsic resistant
