# Supplementary material for: A case for routine microbial diagnostics: Results from antimicrobial susceptibility testing in post-traumatic wound infections at a Ugandan tertiary care hospital
Source: PLOS Glob Public Health. 2023 Aug 15;3(8):e0001880. doi: 10.1371/journal.pgph.0001880 (PMC10427013; doi:10.1371/journal.pgph.0001880)
Supplement: S2 Table — (DOCX) [file pgph.0001880.s002.docx]

S2 Table: Most prevalent pathogens and their resistance towards selected antimicrobials for community acquired infection subgroup

| **Isolates**  **n/N (%)** | ***Acinetobacter* spp.**  **N=14** | ***E. coli***  **N= 12** | ***Klebsiella* spp.**  **N =11** | ***Enterococcus* spp.**  **N= 12** | ***P. aeruginosa***  **N= 9** |
| --- | --- | --- | --- | --- | --- |
| **Ceftriaxone/Cefotaxime^1^** | 7/7 (100.0) | 10/11 (90.9) | 10/10 (100.0) | -^2^ | -^2^ |
| **Gentamicin** | 11/12 (91.7) | 4/9 (44.4) | 9/10 (90.0) | 1/10 (10.0)^3^ | 4/6 (66.7) |
| **Ampicillin** | -^2^ | 9/9 (100.0) | -^5^ | 1/5 (20.0) | -^2^ |
| **Amoxicillin+ clavulanic acid** | -^2^ | 4/9 (44.4) | 3/8 (37.5) | -^2^ | -^2^ |
| **Cefepime** | 11/11 (100.0) | 2/2 (100.0) | 2/2 (100.0)^4^ | -^2^ | -^2^ |
| **Ciprofloxacin** | 8/10 (80.0) | 3/6 (50.0) | 2/10 (20.0) | 2/3 (66.7) | 1/6 (16.7) |
| **Meropenem/Imipenem^1^** | 6/14 (42.9) | 1/12 (8.3) | 2/10 (20.0) | -^2^ | 3/8 (37.5) |
| **Amikacin** | 2/12 (16.7) | -^2^ | 1/2 (50.0) | -^2^ | 3/5 (60.0) |
| **Chloramphenicol** | -^2^ | 2/9 (22.2) | 4/10 (40.0) | 2/8 (25.0) | -^2^ |
| **Piperacillin/**  **Tazobactam** | 9/11 (81.8) | 3/6 (50.0) | 3/6 (50.0) | -^2^ | 2/6 (33.3) |

^1^ One out of two was set

^2^ Intrinsic resistance, not tested

^3^ High-level gentamicin resistance
^4^ Set in case of resistance to 3rd generation cephalosporins

^5^ Intrinsic resistant
